# Prevalence, bacterial etiology, and risk factors of clinical and subclinical mastitis in dromedary camels (*Camelus dromedarius*) across pastoral and peri-urban production systems in south-central Somalia

**DOI:** 10.1186/s12917-026-05792-2

**Published:** 2026-08-04

**Authors:** Shafii Abdullahi Mohamed, Daha Hussein Mumin, Anfac Abdullahu Guled, Abdillahi Jama Abdi, Ibrahim Abdullahi Nur, Hussein Mohamed Farah, Hassan Mohamed Hassan

**Affiliations:** 1 Centre for Excellence in Camel Research & Development (CE-CaRD), Somali National University, Gahayr Campus, Afgoi Road, KM 7, Mogadishu, Somalia; 2 Faculty of Veterinary Medicine and Animal Husbandry, Somali National University (SNU), P.O. Box 15, Mogadishu, Somalia; 3 Department of Biology, Ecology and Earth Sciences (DiBEST), University of Calabria, Via P. Bucci 4C–6C, Arcavacata, Rende, Cosenza 87036 Italy; 4 Department of Veterinary Medicine and Animal Production, University of Naples Federico II, Naples, 80137 Italy

**Keywords:** Camel mastitis, *Camelus dromedarius*, Pastoral systems, Peri-urban dairy, Somatic cell count, Bacterial pathogens, Antibiotic residues, Public health, Somalia

## Abstract

Mastitis is a major constraint to camel milk productivity, udder health, and milk safety in pastoral and emerging peri-urban dairy systems, yet comparative epidemiological data across these systems remain scarce. This cross-sectional study investigated mastitis in lactating dromedary camels in Banadir and Lower Shabelle, south-central Somalia, and examined the relationship between California Mastitis Test (CMT) score and somatic cell count (SCC), together with antibiotic residues in a subset of milk samples. Between August 2024 and January 2026, 384 lactating camels were enrolled, yielding 1,536 quarter milk samples. Active clinical mastitis was defined by visible udder or milk abnormalities at examination; CMT positivity was reported independently of clinical status. SCC was measured in 1,419 quarter samples, and bacteriological culture was performed on all quarter milk samples. At camel level, 48.4% had at least one quarter with active clinical signs, 91.7% had at least one CMT-positive quarter, and 68.2% had at least one culture-positive quarter. At quarter level, clinical mastitis, CMT positivity, and culture positivity were 16.5%, 47.8%, and 24.6%, respectively. Coagulase-negative staphylococci were the most frequent isolates (28.5%), followed by *Staphylococcus aureus* (22.0%), *Escherichia coli* (13.2%), *Streptococcus agalactiae* (11.0%), and *Streptococcus uberis* (9.9%). Median SCC increased from 99,548 cells/mL in CMT score 0 quarters to 2,542,414 cells/mL in CMT score 3 quarters (*p* < 0.001). In multivariable generalized estimating equation analysis, peri-urban production system (AOR = 1.53; 95% CI: 1.24–1.89), mild teat lesions (AOR = 1.54; 95% CI: 1.12–2.10), and severe teat lesions (AOR = 2.02; 95% CI: 1.28–3.18) were significantly associated with quarter-level CMT positivity. Of 60 samples subjected to TriSensor testing, 18 (30.0%) were positive; beta-lactams were the most frequent residue group (10/18; 55.6%). These findings show that mastitis is widespread in south-central Somali camel dairying and is shaped by bacterial infection and modifiable management-related factors. Improved udder-health surveillance, teat-health management, and prudent antimicrobial-use practices are needed to reduce disease burden and improve camel milk safety.

**Supplementary Information:**

The online version contains supplementary material available at https://doi.org/10.1186/s12917-026-05792-2.

## Introduction

Somalia is one of the world’s major livestock-dependent countries, where pastoral and agro-pastoral systems operate under harsh dryland conditions and milk production remains central to food security and rural livelihoods [1–4]. National estimates have placed the Somali camel population at approximately seven million head [1]. Globally, dromedary camels support food security and pastoral livelihoods across arid, semi-arid, and desert regions of Africa and Asia by providing milk, meat, transport, and income where other livestock production is constrained [5, 6]. This role is underpinned by their adaptation to drought, high temperatures, erratic feed resources, and long-distance mobility, which enables them to maintain milk production under conditions that constrain other dairy species [1, 3, 7]. In Somalia and across the wider Horn of Africa, camel milk supports household nutrition, income generation, and livelihood resilience, while commercialization and urban demand are increasing the importance of camel dairying in both pastoral and peri-urban settings [8, 9]. However, expansion of the camel milk sector has also intensified concerns around hygienic milking, veterinary support, informal marketing, and milk safety, particularly where cold-chain infrastructure and quality assurance remain limited [2, 3, 10, 11]. At the production level, inadequate feed and water availability, unsuitable housing conditions, and gaps in preventive healthcare may also compromise camel welfare; recent assessment under pastoral conditions identified feeding and housing as important areas of concern [7].

Within this context, udder health is a key determinant of both productivity and milk safety. Mastitis, defined as inflammation of the mammary gland, is one of the most important health constraints in dairy animals and is increasingly recognized as a major problem in camels [10, 12, 13]. Regional studies have reported camel-level mastitis prevalence ranging from 22.4% in southern Ethiopia to 59.8% in the Afar Region, illustrating substantial variation across settings [14, 15]. Mastitis occurs in both clinical and subclinical forms. Clinical mastitis is characterized by visible abnormalities of the udder or milk, whereas subclinical mastitis is not readily apparent without screening and may persist while reducing milk yield, altering milk composition, and facilitating continued pathogen shedding [10, 12]. In camel production systems, where hand-milking, calf-at-foot management, limited routine laboratory testing, and informal milk marketing are common, subclinical infection may remain undetected while still carrying biological, economic, and public-health consequences [8, 10, 13].

Camel mastitis is important not only because of milk loss, but also because intramammary infection affects milk quality and safety. Mastitis has been associated with elevated somatic cell counts (SCC), altered physicochemical characteristics of milk, and recovery of important bacterial pathogens [9, 10]. Studies in camel dairies have shown that SCC varies with udder health status and management conditions, supporting its value as an indicator of mammary inflammation and milk-quality deterioration [9, 11, 16]. Because healthy baseline distributions and proposed diagnostic thresholds in dromedaries differ from bovine standards, camel SCC requires species-specific and context-dependent interpretation [8, 17]. A wide range of bacterial pathogens has been implicated, including *Staphylococcus aureus*, coagulase-negative staphylococci, streptococci, *Escherichia coli*, and other contagious or environmental organisms, although their relative importance varies across production systems and settings [10, 14, 18–22]. This mixed contagious-environmental pattern implies that effective control depends on both improved milking hygiene and management of environmental exposure [10, 13].

Milk-safety concerns are especially relevant in camel systems because milk is often consumed raw or only minimally processed in pastoral and peri-urban settings [23, 24]. Under these conditions, mastitis may increase bacterial contamination risk, while treatment can introduce antimicrobial residues when withdrawal periods are poorly understood or not observed [23, 24]. Informal camel milk value chains in East Africa contain hygiene gaps from production to market [18, 23], and camel-specific evidence suggests that some antibiotics may persist in milk longer than expected. Recent experimental work evaluated penicillin, oxytetracycline, and streptomycin and showed class-specific differences in residue depletion from camel milk [24], underscoring the need for species-specific milk-safety data rather than direct extrapolation from cattle.

The epidemiology of camel mastitis is shaped by interacting host, environmental, and management-related factors. Previous studies have identified associations with age, parity, stage of lactation, tick infestation, udder and teat lesions, milking hygiene, and production system [14, 18–20, 22]. Teat and udder lesions are particularly important because they compromise the natural barrier to infection, while production-system differences may influence stocking density, handling intensity, veterinary access, and pathogen transmission dynamics [14, 19, 22]. Diagnostic studies in dromedaries show that CMT scores generally increase with SCC, whereas concordance with bacteriological culture is incomplete; diagnostic thresholds also remain partly context-specific in camels [8, 11, 13]. In field settings where laboratory capacity may be constrained, CMT, SCC, and bacteriology are therefore best used as complementary tools for udder-health surveillance.

Despite the nutritional and economic importance of camels in Somalia, integrated epidemiological evidence on camel mastitis remains limited. Available studies from neighboring regions indicate that mastitis is common, multifactorial, and influenced by production environment, but Somali data remain scarce, particularly for studies that jointly assess clinical mastitis, subclinical inflammation, bacteriological positivity, and residue-related milk-safety concerns across contrasting production systems [14, 19, 20, 22]. This knowledge gap is important because pastoral and peri-urban camel dairying differ markedly in herd mobility, market integration, handling intensity, and management conditions, all of which may shape disease risk and milk quality [2, 3, 23]. Therefore, the present study aimed to estimate clinical mastitis, CMT positivity, and bacteriological culture positivity at camel and quarter levels in lactating dromedary camels across Banadir and Lower Shabelle, south-central Somalia; identify associated risk factors; characterize major bacterial isolates; and assess antibiotic residues in a subset of milk samples.

## Methods

### Study design and setting

This cross-sectional study was conducted between August 2024 and January 2026 in Banadir and Lower Shabelle, south-central Somalia. Six districts represented two camel production systems: pastoral (Afgooye, Walanweyn, and Yaaqbariweyne in Lower Shabelle) and peri-urban (Dharkenley, Garasbaley, and Kaxda in Banadir).

### Study population and sample size

The study population comprised lactating one-humped camels (*Camelus dromedarius*) managed under the selected pastoral and peri-urban production systems. Camels were eligible if they were lactating, had four accessible udder quarters, and could be safely restrained for clinical examination and milk sampling. Herds and individual camels were selected using simple random sampling within the study districts.

Sample size was determined using Cochran’s formula for prevalence studies:

$$\:n=\frac{{Z}^{2}p\left(1-p\right)}{{d}^{2}}$$

where *n* is the required sample size, *Z* is the standard normal deviate at the 95% confidence level, *p* is the expected prevalence, and *d* is the desired precision. Because no reliable baseline prevalence estimate was available for the exact Somali districts studied, an expected prevalence of 50% was selected to maximize the required sample size. With 5% precision, the minimum sample size was 384 camels [25]. Accordingly, 384 lactating camels were enrolled, yielding 1,536 quarter-level observations.

### Clinical examination and milk sampling

Field data were recorded using a structured case-record form.Recorded camel-level variables included district, production system, owner-reported age, parity, body condition score, lactation stage, breed or ecotype, and recent antibiotic exposure when treatment history was available. Lactation stage, breed or ecotype, and recent antibiotic exposure were excluded from final analyses because the corresponding field records were incomplete or not sufficiently standardized across herds. Because owner-reported age data included biologically implausible values and could not be verified from birth records, age was excluded from the final descriptive and regression analyses. Before milk collection, each camel underwent clinical examination of the udder and teats. Quarter-level observations included quarter position, teat lesion severity, and tick severity.

Milk samples were collected aseptically from all four udder quarters (left fore, right fore, left hind, and right hind). Teat ends were cleaned and disinfected with 70% alcohol, the first streams of milk were discarded, and approximately 10–15 mL of milk was collected into sterile labelled tubes. Samples were kept chilled during handling and transport to the Somali National University Diagnostic Veterinary Laboratory for analysis.

### California mastitis test

All 1,536 quarter milk samples were tested using the California Mastitis Test (CMT) at the Somali National University Diagnostic Veterinary Laboratory, following the standard method described by ^24^. Approximately 2 mL of milk was mixed with an equal volume of commercial CMT reagent in a paddle cup and rotated gently for 10–15 s. Reactions were scored as 0, Trace, 1, 2, or 3 according to gel formation. Quarters with CMT scores of 1 or greater were classified as positive, whereas Trace was considered negative, in line with established interpretation criteria [26–28]. The reagent used was WIKO^®^ California Mastitis Test (Gluetec Industrial Adhesives, Germany).

### Somatic cell count

Somatic cell count (SCC) was measured for 1,419 of the 1,536 quarter milk samples at the Somali National University Diagnostic Veterinary Laboratory using a Fossomatic™ 7 somatic cell counter (FOSS, Hillerød, Denmark), based on the fluoro-opto-electronic method. The remaining 117 samples had insufficient residual volume or failed instrument quality-control criteria and were excluded from SCC-specific analyses. SCC was evaluated as an indicator of mammary inflammation in relation to CMT score categories [29, 30].

### Bacteriological analysis and isolate identification

Bacteriological culture was performed on all 1,536 quarter milk samples, and isolate identification was conducted at the Somali National University Diagnostic Veterinary Laboratory. Milk aliquots were cultured on 5% sheep blood agar and MacConkey agar and incubated aerobically at 35–37 °C. Plates were examined after 18–24 h and, when necessary, again at 48 h. Bacterial isolates were characterized on the basis of colony morphology, haemolysis, lactose fermentation, Gram reaction, and routine biochemical characteristics. Samples showing mixed, non-interpretable growth of unrelated colony types were classified as contaminated rather than as true intramammary infection.

Presumptive identification was further supported by matrix-assisted laser desorption/ionization time-of-flight mass spectrometry (MALDI-TOF MS).

### Antibiotic residue screening

Antibiotic residue screening was conducted at the Somali National University Diagnostic Veterinary Laboratory on camel-level milk samples. Of 384 samples available for this component, 360 were screened using Delvotest^®^ (dsm-firmenich, Delft, the Netherlands); 24 were excluded because the milk was spoiled or of insufficient volume. A subset of 60 Delvotest^®^-positive or unclear samples with adequate remaining volume was subsequently analysed using TriSensor^®^ (Unisensor, Seraing, Belgium), a rapid dipstick assay for beta-lactams, sulfonamides, and tetracyclines in milk.

### Outcome definitions

The primary outcomes were active clinical mastitis, quarter-level CMT positivity, and quarter-level bacteriological culture positivity. Active clinical mastitis was defined by visible abnormalities present at examination. Udder abnormalities included swelling, heat, pain, or marked asymmetry, while milk abnormalities included clots, flakes, watery secretion, or discoloration. Historical episodes without current visible signs were not classified as active clinical mastitis. A quarter was CMT-positive if its score was at least 1; CMT positivity was classified independently of clinical status and was therefore not synonymous with exclusively subclinical disease. A quarter was culture-positive when growth was consistent with intramammary infection. At camel level, an animal was classified as positive if at least one quarter met the relevant outcome definition.

### Statistical analysis

Data were analysed in R. Categorical variables were summarized as frequencies and percentages. Because SCC data were right-skewed, they were summarized using medians and interquartile ranges (IQR), and differences across CMT score categories were assessed using the Kruskal-Wallis test [31].

Associations between quarter-level CMT positivity and categorical explanatory variables were first evaluated using Pearson’s chi-square tests; all expected cell counts for Table 1 comparisons exceeded 5. Variables associated in univariable analysis at *p* < 0.20, together with variables considered epidemiologically important, were entered into a multivariable generalized estimating equation (GEE) logistic regression model with a logit link and exchangeable working correlation structure. The final model simultaneously included production system, teat lesion severity, tick severity, and quarter position, with camel identity as the clustering variable [32]. Adjusted odds ratios (AOR) and 95% confidence intervals (CI) were reported. All tests were two-sided, and *p* < 0.05 was considered statistically significant.

**Table 1:** Study population characteristics by production system (*n* = 384 camels)

Variable	Category	Overall *n* (%)	Pastoral *n* (%)	Peri-urban *n* (%)	*p*-value
Parity	1 calving	126 (32.8)	73 (32.7)	53 (32.9)	0.360
	2–3 calvings	121 (31.5)	76 (34.1)	45 (28.0)	
	≥ 4 calvings	137 (35.7)	74 (33.2)	63 (39.1)	
Body condition score	≤ 2.5 (poor)	124 (32.3)	100 (44.8)	24 (14.9)	< 0.001
	3.0 (moderate)	129 (33.6)	74 (33.2)	55 (34.2)	
	≥ 3.5 (good)	131 (34.1)	49 (22.0)	82 (50.9)	

Age rows were excluded because owner-reported values contained biologically implausible observations that could not be verified. *p*-values are from Pearson’s chi-square tests; all expected cell counts exceeded 5

## Results

### Study population characteristics

A total of 384 lactating dromedary camels were enrolled from pastoral and peri-urban production systems in south-central Somalia, contributing 1,536 quarter milk samples. Of these, 223 (58.1%) camels were from pastoral herds and 161 (41.9%) from peri-urban herds. Poor body condition (≤ 2.5) was more common in pastoral camels (44.8%) than in peri-urban camels (14.9%), whereas good body condition (≥ 3.5) was more frequent in peri-urban camels (50.9%) than in pastoral camels (22.0%) (*p* < 0.001). Parity distributions did not differ significantly between production systems (Table 1).

Lactation stage, breed or ecotype, and recent antibiotic-exposure fields were not summarized or modelled because their records were incomplete or not sufficiently standardized across herds.

### Clinical, subclinical, and bacteriological mastitis outcomes

**Fig. 1:**
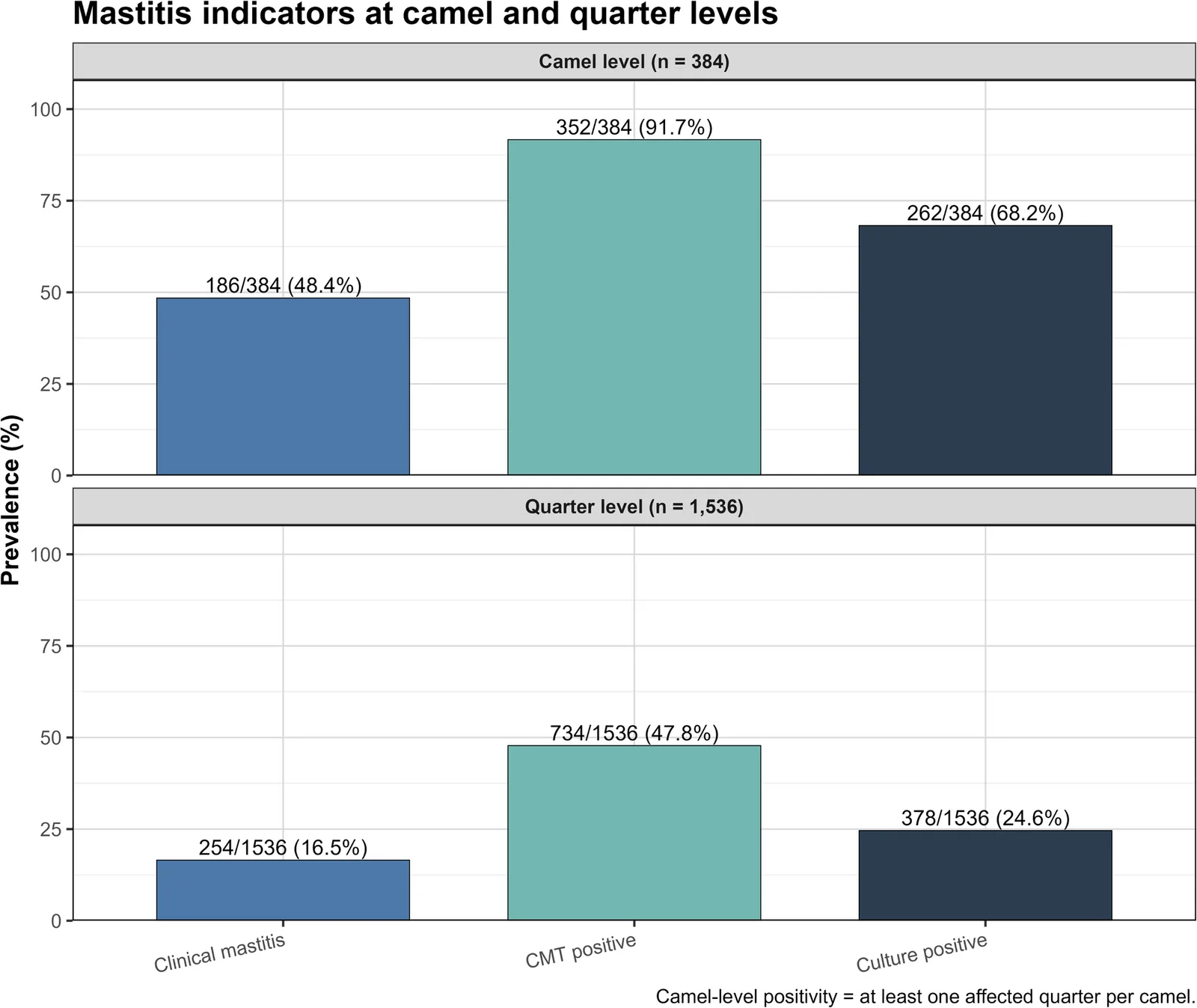
Prevalence of mastitis indicators at camel and quarter levels in lactating dromedary camels in south-central Somalia. Camel-level prevalence was defined as at least one affected quarter per camel; quarter-level prevalence used all examined quarters. CMT, California Mastitis Test

At camel level, 186/384 camels (48.4%) had at least one quarter with active clinical mastitis, 352/384 (91.7%) had at least one CMT-positive quarter, and 262/384 (68.2%) had at least one culture-positive quarter. Camel-level CMT positivity was defined independently of clinical status. At quarter level, 254/1,536 quarters (16.5%) had active clinical mastitis, 734/1,536 (47.8%) were CMT-positive, and 378/1,536 (24.6%) were culture-positive. The higher camel-level clinical prevalence reflects aggregation across four quarters, because one clinically affected quarter classified the entire camel as positive. By quarter, CMT positivity ranged from 44.8% in the right fore quarter to 50.3% in the right hind quarter, while culture positivity ranged from 22.1% in the right fore quarter to 26.8% in the left hind quarter (Table 2; Fig. 1).

**Table 2:** Mastitis indicators at camel and quarter levels

Level	Measure	*n*	Clinical mastitis *n* (%)	CMT positive *n* (%)	Culture positive *n* (%)
Camel	Any quarter with active signs	384	186 (48.4)	352 (91.7)	262 (68.2)
Quarter	Left fore	384	68 (17.7)	183 (47.7)	92 (24.0)
Quarter	Right fore	384	48 (12.5)	172 (44.8)	85 (22.1)
Quarter	Left hind	384	67 (17.4)	186 (48.4)	103 (26.8)
Quarter	Right hind	384	71 (18.5)	193 (50.3)	98 (25.5)
Quarter overall	All quarters	1,536	254 (16.5)	734 (47.8)	378 (24.6)

Camel-level CMT positivity was defined independently of clinical status; a camel was positive when at least one quarter met the relevant outcome definition

**Fig. 2:**
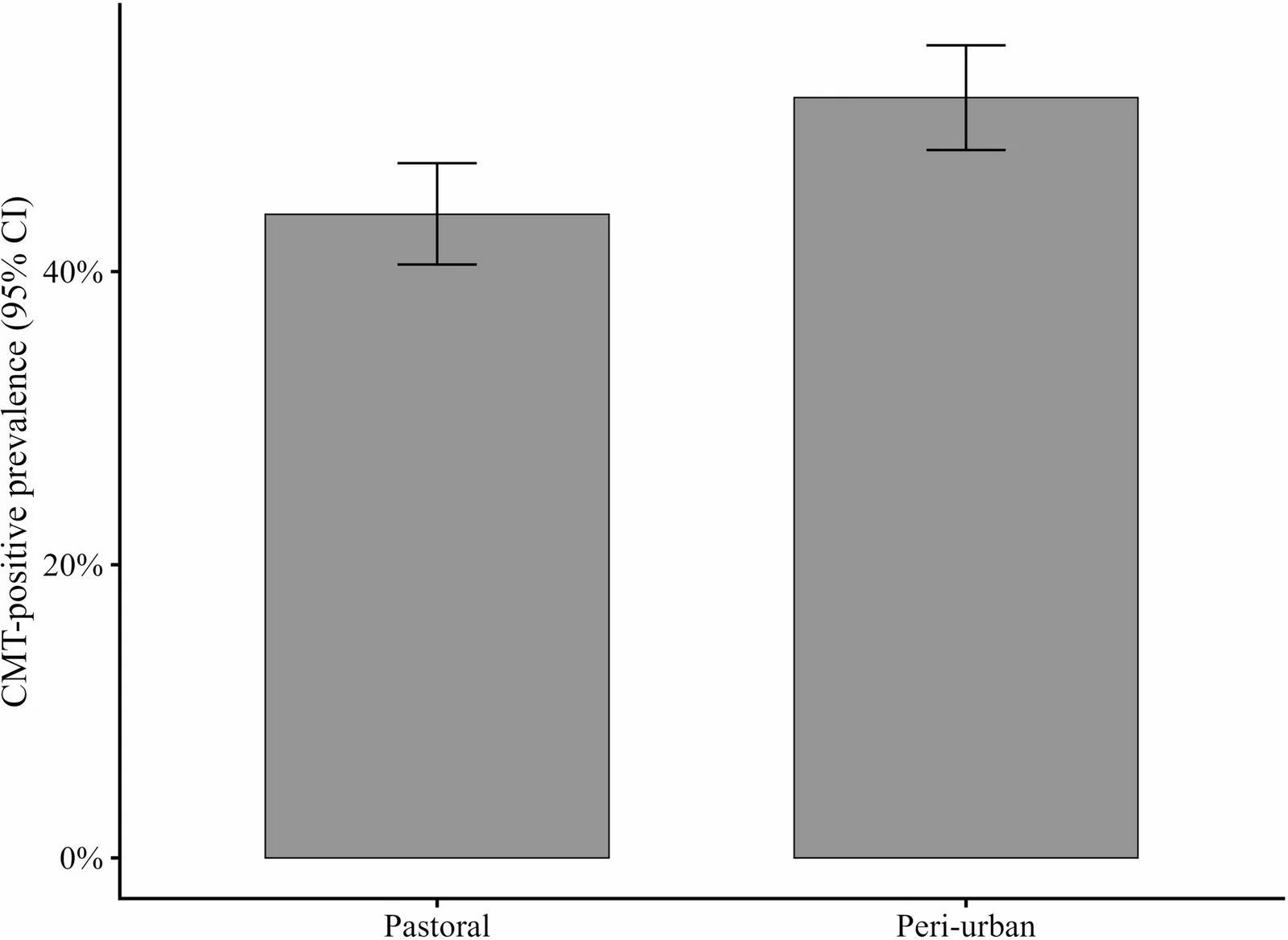
Quarter-level prevalence of CMT positivity by production system. Bars show the proportion of quarter milk samples classified as CMT-positive in pastoral and peri-urban production systems. CMT positivity was defined as a score of at least 1

Quarter-level CMT positivity was higher in peri-urban than in pastoral production systems in the stratified comparison shown in Fig. 2 (Fig. 2).

### Bacterial isolates recovered from culture-positive milk samples

**Fig. 3:**
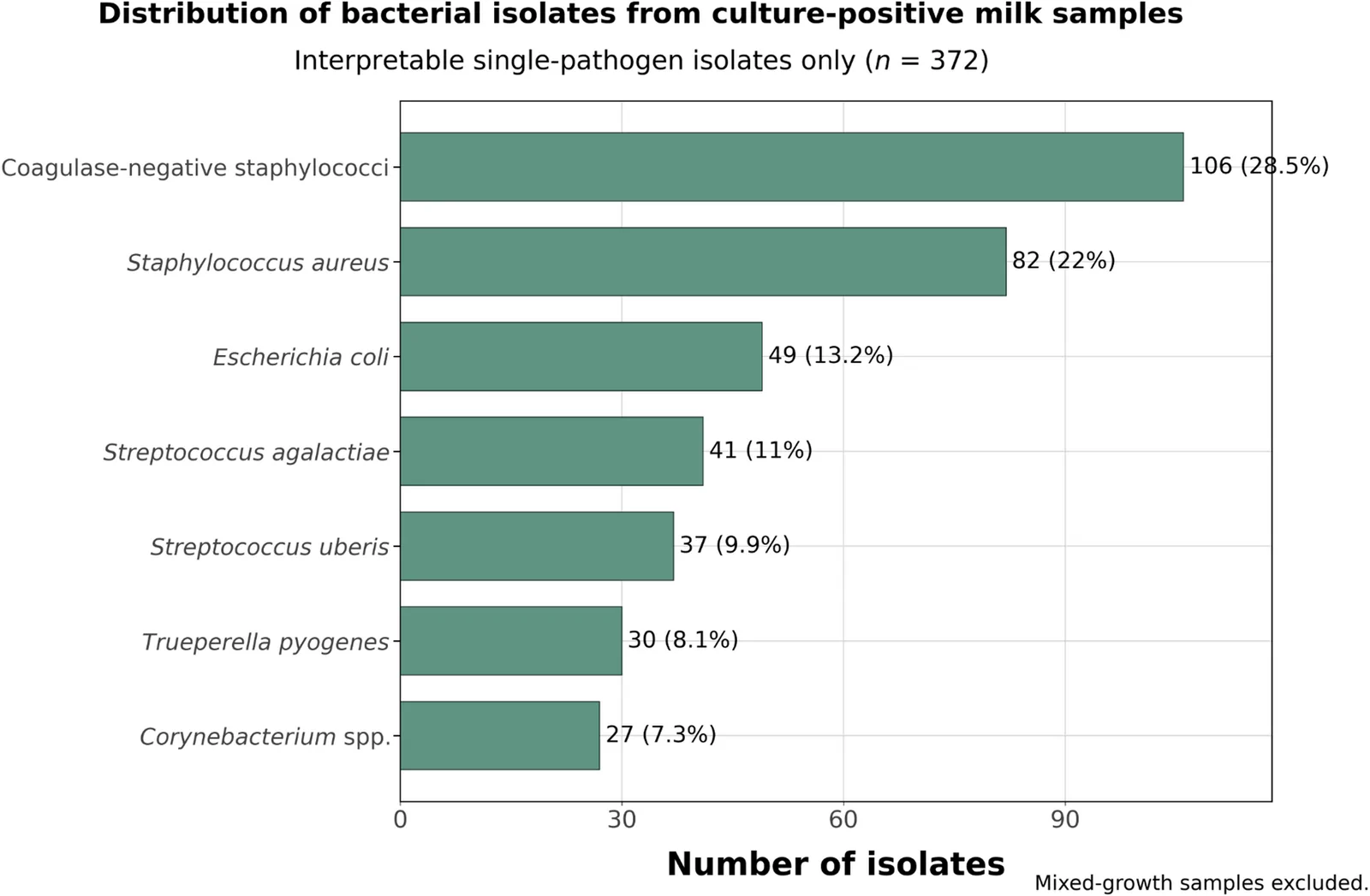
Distribution of bacterial isolates recovered from culture-positive quarter milk samples. The figure presents the frequency distribution of major bacterial pathogens isolated from culture-positive milk samples. Percentages are based on interpretable single-pathogen isolates; samples with mixed non-interpretable growth were excluded from the pathogen distribution summary

Among 372 interpretable single-pathogen isolates, coagulase-negative staphylococci (106; 28.5%), *Staphylococcus aureus* (82; 22.0%), and *Escherichia coli* (49; 13.2%) were the three most frequent groups. The complete distribution is presented in Table 3; Fig. 3. Six of the 378 culture-positive samples had mixed growth and were excluded from the single-pathogen distribution.

**Table 3:** Distribution of interpretable single-pathogen isolates from culture-positive milk samples (*n* = 372)

Pathogen	*n*	%
Coagulase-negative staphylococci	106	28.5
*Staphylococcus aureus*	82	22.0
*Escherichia coli*	49	13.2
*Streptococcus agalactiae*	41	11.0
*Streptococcus uberis*	37	9.9
*Trueperella pyogenes*	30	8.1
*Corynebacterium* spp.	27	7.3

Six of 378 culture-positive samples had mixed growth and were excluded. Percentages use the 372 interpretable single-pathogen isolates as the denominator

### Somatic cell count according to CMT category

**Fig. 4:**
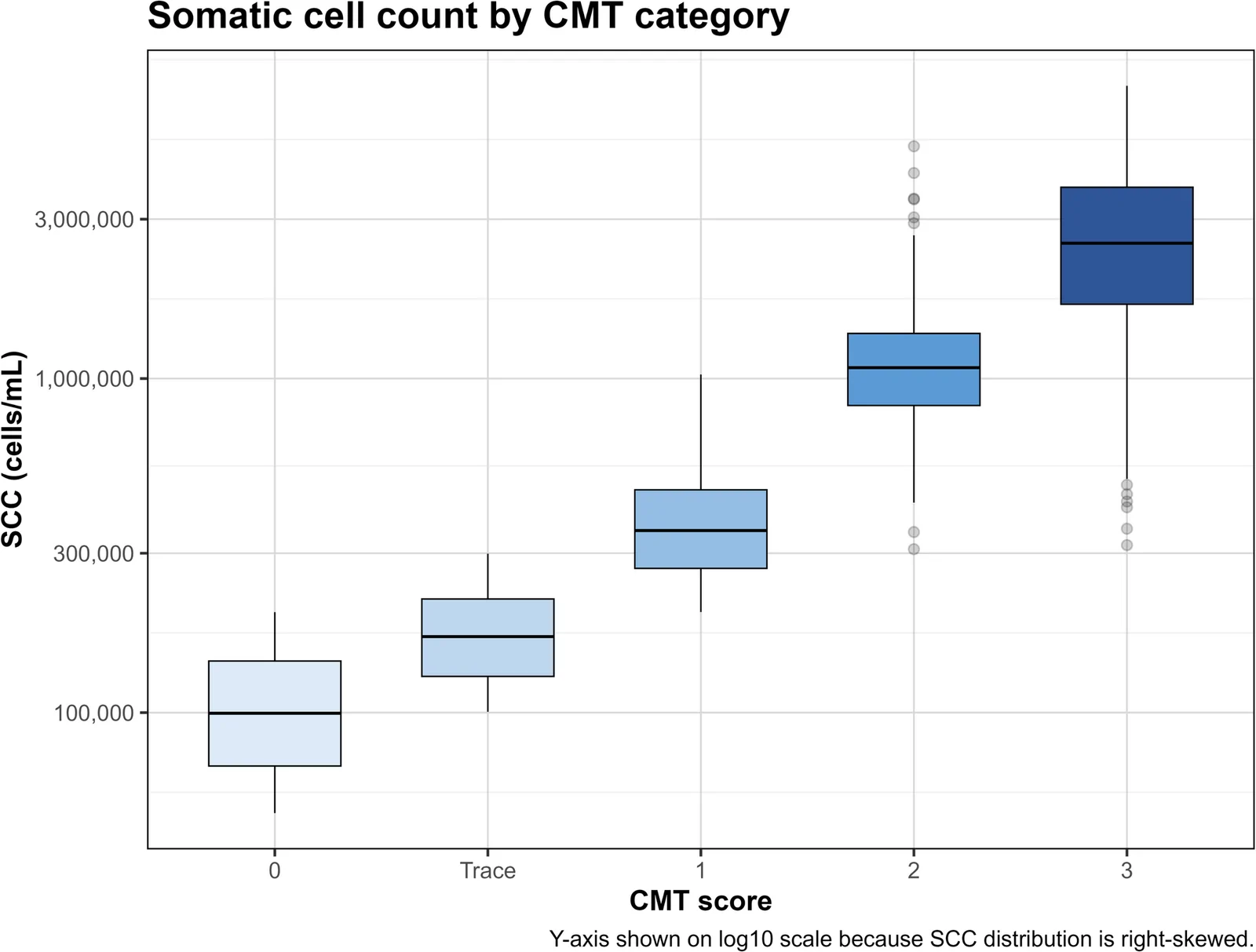
Somatic cell count distribution across California Mastitis Test categories. The figure illustrates somatic cell count (SCC) values across CMT score categories (0, Trace, 1, 2, and 3) among quarter milk samples with SCC measurements. SCC increased progressively with increasing CMT score. CMT, California Mastitis Test; SCC, somatic cell count

Somatic cell count increased progressively across CMT categories. Among 1,419 quarters with SCC measurements, median SCC was 99,548 cells/mL (IQR: 69,212–142,720) for CMT score 0, 168,872 cells/mL (128,358–218,897) for trace, 350,753 cells/mL (270,270–464,538) for score 1, 1,077,864 cells/mL (830,662–1,364,948) for score 2, and 2,542,414 cells/mL (1,669,050–3,739,685) for score 3. The increase from score 1 to score 2 crossed one million cells/mL, identifying CMT score 2 as a practical field signal of marked mammary inflammation in this dataset. Differences across CMT categories were significant (Kruskal-Wallis, *p* < 0.001) (Table 4; Fig. 4).

**Table 4:** Somatic cell count by CMT category (*n* = 1,419 quarters with SCC measured)

CMT score	*n*	Median SCC (IQR), cells/mL
0	522	99,548 (69,212 − 142,720)
Trace	218	168,872 (128,358 − 218,897)
1	295	350,753 (270,270–464,538)
2	180	1,077,864 (830,662-1,364,948)
3	204	2,542,414 (1,669,050 − 3,739,685)

### Factors associated with quarter-level CMT positivity

**Fig. 5:**
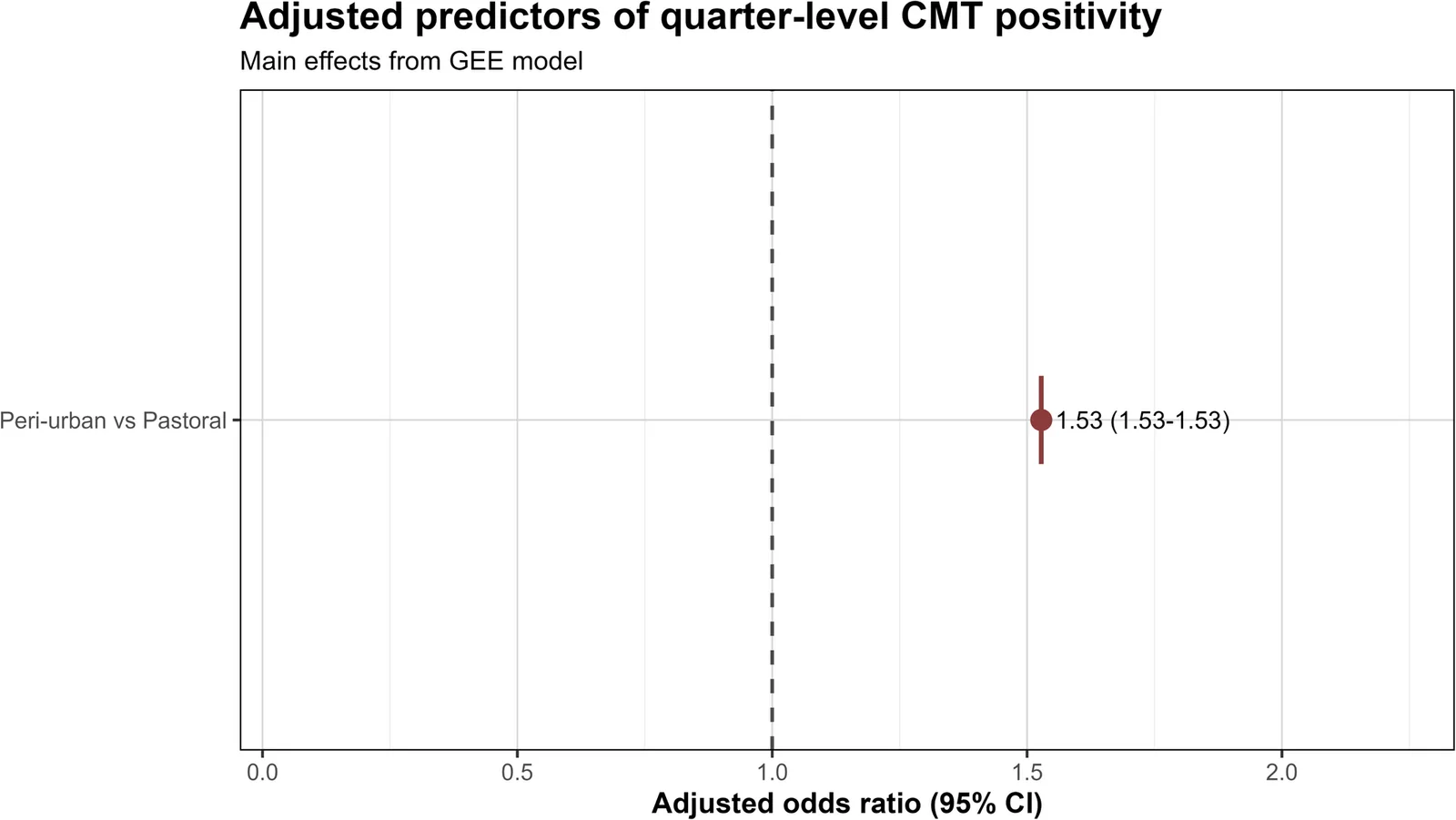
Adjusted predictors of quarter-level CMT positivity in the generalized estimating equation model. The figure shows adjusted odds ratios and 95% confidence intervals for variables retained in the multivariable GEE logistic regression model assessing quarter-level CMT positivity. The model accounted for clustering of quarters within camels. AOR, adjusted odds ratio; CI, confidence interval; CMT, California Mastitis Test; GEE, generalized estimating equation

In the multivariable GEE logistic regression model, quarters from peri-urban camels had higher odds of CMT positivity than quarters from pastoral camels (AOR = 1.53; 95% CI: 1.24–1.89; *p* < 0.001). Compared with quarters without teat lesions, mild lesions (AOR = 1.54; 95% CI: 1.12–2.10; *p* = 0.008) and severe lesions (AOR = 2.02; 95% CI: 1.28–3.18; *p* = 0.003) were associated with higher odds of CMT positivity. Moderate lesions were not significantly associated (AOR = 1.11; 95% CI: 0.85–1.44; *p* = 0.448). Tick severity and quarter position were not significantly associated with the outcome (Table 5; Fig. 5).

**Table 5:** Multivariable generalized estimating equation logistic regression for quarter-level CMT positivity

Variable	Category	AOR (95% CI)	*p*-value
Production system (ref: Pastoral)	Peri-urban	1.53 (1.24–1.89)	< 0.001
Teat lesion severity (ref: None)	Mild	1.54 (1.12–2.10)	0.008
	Moderate	1.11 (0.85–1.44)	0.448
	Severe	2.02 (1.28–3.18)	0.003
Tick severity (ref: None)	Mild	0.89 (0.70–1.14)	0.347
	Moderate	1.19 (0.91–1.56)	0.206
	Severe	0.86 (0.63–1.18)	0.362
Quarter sampled (ref: LF)	RF	0.89 (0.66–1.20)	0.438
	LH	1.03 (0.78–1.37)	0.825
	RH	1.11 (0.83–1.50)	0.484

*AOR* adjusted odds ratio, *CI* confidence interval, *LF* left fore, *RF* right fore, *LH* left hind, *RH* right hind

### Antibiotic residue screening

Of 384 camel-level milk samples available for residue analysis, 24 were spoiled or insufficient and 360 were screened using Delvotest^®^. Among screened samples, 270/360 (75.0%) were negative, 56/360 (15.6%) were positive, and 34/360 (9.4%) were unclear. A subset of 60 Delvotest^®^-positive or unclear samples was tested using TriSensor^®^: 18/60 (30.0%) were positive, 36/60 (60.0%) were negative, and 6/60 (10.0%) were unclear. Among the 18 TriSensor-positive samples, beta-lactams accounted for 10 (55.6%), sulfonamides for 4 (22.2%), tetracyclines for 2 (11.1%), and beta-lactams plus tetracyclines for 2 (11.1%). These 18 samples represented 5.0% of all 360 Delvotest-screened samples on a simple denominator basis; because TriSensor testing was restricted to a selected subset, this is descriptive and not a population prevalence estimate (Tables 6 and 7).

**Table 6:** Number of screening tests and results of Delvotest^®^ by production system and district

Result / test step	Total	Pastoral total	Afgooye	Walan weyn	Yaaqbariweyne	Peri-urban total	Dharkenley	Garasbaley	Kaxda
Samples (milk samples collected)	384	223	90	66	67	161	64	48	49
Delvotest^®^ (tested)	360	209	84	62	63	151	61	45	45
Negative	270	170	68	50	52	100	41	30	29
Positive	56	21	9	6	6	35	14	10	11
Unclear	34	18	7	6	5	16	6	5	5
TriSensor^®^ (subset tested)	60	20	8	6	6	40	16	12	12

Twenty-four of 384 camel-level milk samples (14 pastoral and 10 peri-urban) were excluded from Delvotest^®^ screening because they were spoiled or of insufficient volume

**Table 7:** Screening test results by TriSensor^®^

Antibiotic residue group (TriSensor^®^)	TriSensor-positive, *n*	Among TriSensor-positive samples, %	Among all Delvotest^®^-screened samples (*n* = 360), %	TriSensor-unclear, *n*
All TriSensor results	18	100.0	5.0	6
Beta-lactams	10	55.6	2.8	3
Tetracyclines	2	11.1	0.6	0
Sulfonamides	4	22.2	1.1	0
Beta-lactams + tetracyclines	2	11.1	0.6	2
Beta-lactams + tetracyclines + sulfonamides	0	0.0	0.0	1

Percentages in the fourth column use all Delvotest^®^-screened samples (*n* = 360) as the denominator. Because TriSensor^®^ testing was restricted to a selected subset of 60 samples, these percentages are descriptive and are not population prevalence estimates

## Discussion

### Burden of mastitis across production systems

This study demonstrates a high burden of udder disease in lactating dromedary camels in south-central Somalia. At camel level, 48.4% had at least one quarter with active clinical mastitis. This exceeded the 4.3% camel-level clinical prevalence reported in Gomole District, southern Ethiopia, where total camel-level mastitis prevalence was 22.4% and quarter-level mastitis prevalence was 16.6%^17^. Direct comparisons require caution because outcome definitions and units of analysis differ. In the present study, classifying a camel as positive when any of four quarters showed active signs partly explains the higher camel-level estimate. Herds were sampled within study districts and were not recruited because of suspected active outbreaks. The much higher frequency of CMT-positive than clinically affected quarters indicates that subclinical inflammation remains a major hidden component of disease burden. These findings are consistent with studies identifying mastitis as an important constraint to camel milk production and udder health [10, 13–15, 18, 33].

Within Somalia, a previous Benadir study reported 9.85% camel-level subclinical mastitis [34], while CMT-based work around Hargeisa reported 24.7% camel-level mastitis [35]. In Isiolo County, Kenya, 46% of camels and 26% of quarters met a stricter subclinical definition, and teat or udder skin lesions were similarly identified as a risk factor [13]. In Jigjiga City, Somali Region of Ethiopia, camel-level subclinical prevalence was 10.6%, with poor udder and leg hygiene associated with CMT positivity [21]. These estimates place the present burden toward the upper end of regional reports, but differences in case definitions, CMT thresholds, denominators, and production systems preclude direct ranking.

### Bacteriological profile and epidemiological interpretation

The isolate profile was dominated by coagulase-negative staphylococci and *Staphylococcus aureus*, with additional recovery of *Escherichia coli*, streptococci, *Trueperella pyogenes*, and *Corynebacterium* spp. This pattern is consistent with reports from camel systems in Ethiopia, Kenya, and elsewhere in which Gram-positive cocci predominate while environmental pathogens also contribute [10, 14, 18–20, 36]. Coagulase-negative staphylococci can form part of opportunistic teat-skin microbiota; their prominence is therefore epidemiologically consistent with the observed association between teat lesions and CMT positivity, although isolate source was not directly characterized in this study [10, 13]. Overall, the profile suggests both contagious and environmental transmission and supports combined attention to milking hygiene, teat health, and milking-environment cleanliness [10, 12, 13].

### Diagnostic interpretation of CMT and somatic cell count

A major internal strength of the study was the clear progressive increase in SCC across CMT categories. This stepwise gradient supports the biological usefulness of CMT as a practical indicator of mammary inflammation in Somali camels and is consistent with earlier studies reporting positive relationships among CMT reactivity, SCC, and mastitis status in dromedaries [8, 9, 11, 17]. The finding also accords with broader mastitis literature showing that indirect screening tests reflect inflammatory response rather than direct bacterial recovery alone [12, 30].

In the present study, CMT positivity was substantially more frequent than culture positivity. CMT detects inflammatory change, whereas culture depends on recovery of viable organisms at sampling. Positive CMT reactions may therefore occur with low bacterial load, intermittent shedding, partial resolution, or non-bacterial irritation, while culture may underestimate infection because of viability loss, transport conditions, or previous antimicrobial exposure [10, 12, 30]. Camel milk also contains natural antimicrobial factors, including lysozyme, lactoferrin, and immunoglobulins, which may inhibit bacterial growth in vitro and contribute to culture-negative results despite inflammation [3, 10]. This mechanism was not measured here and should be interpreted as a plausible additional explanation. The findings support use of CMT, SCC, and bacteriology as complementary rather than interchangeable tools.

### Factors associated with quarter-level CMT positivity

The multivariable analysis identified peri-urban production system and teat lesion severity as principal predictors of quarter-level CMT positivity, pointing to modifiable management risk. Damage to teat skin or the teat canal can weaken the udder’s natural defense barrier, facilitate bacterial entry, and sustain inflammation [10, 14, 22, 36]. The lesion estimates were not strictly monotonic: mild and severe categories were significant, whereas the moderate category was not. This pattern may reflect sampling variability, sparse observations within severity strata, or field misclassification. Categories were retained to avoid post hoc recategorization, and the moderate estimate should be interpreted cautiously rather than as evidence of a biological dose-response.

The higher odds of CMT positivity in peri-urban herds are important. Peri-urban camel dairying is often more market-oriented and may involve more frequent handling, closer confinement, repeated equipment contact, and pressure for regular milk offtake. Where hygiene and udder-health monitoring do not improve with intensification, these conditions may increase mastitis risk [2, 3, 16, 23, 37]. Parity was not a dominant predictor in the final model. This may reflect relatively homogeneous exposure pressures, imprecision in owner-reported histories, or limited contrast among parity strata; it does not exclude an effect in other populations and should not be interpreted as contradicting previous camel or bovine evidence [10, 13, 14, 19].

### Antibiotic residues and milk-safety implications

The residue-screening component identified Delvotest^®^ positive or unclear reactions in a notable proportion of samples, and TriSensor^®^ detected residues in a selected subset, with beta-lactams predominating. Because only selected Delvotest^®^-positive or unclear samples underwent TriSensor^®^ testing, the results demonstrate residue occurrence but do not estimate population prevalence. Even so, confirmed group-specific screening reactions indicate that antimicrobial exposure and withdrawal compliance are relevant concerns in the Somali camel milk chain, where milk may be consumed raw or minimally processed [2, 3, 23, 38]. Withdrawal periods cannot be transferred directly from cattle because species-specific absorption, distribution, metabolism, elimination, and milk composition can alter drug depletion. Recent camel experiments with penicillin, oxytetracycline, and streptomycin demonstrated drug-specific residue kinetics [24]. These findings support integrated mastitis control, prudent antimicrobial use, and milk-quality assurance.

### Strengths and limitations

This study has several strengths. It included a relatively large sample of lactating camels, quarter-level assessment of all four udder quarters, and a multi-method design combining clinical examination, CMT, SCC, bacteriology, and ancillary residue screening. The use of GEE modelling strengthened inference by accounting for clustering of quarters within camels. In addition, the study provides rare comparative evidence from pastoral and peri-urban camel production systems in Somalia, where integrated mastitis epidemiology remains poorly documented [10, 13, 14].

Some limitations should also be recognized. The cross-sectional design limits causal inference. Culture-based diagnosis may have underestimated infection in some inflamed quarters, and the selected CMT threshold may have increased sensitivity to inflammatory change relative to bacteriological confirmation. Owner-reported age data were excluded because implausible values could not be verified, and 117 quarters lacked valid SCC results. The residue analysis was descriptive and based on selected subset testing rather than confirmatory testing of all samples. Nevertheless, consistency across clinical examination, CMT, SCC, bacteriology, and regression findings supports the conclusion that mastitis is widespread and management-relevant in the study area.

## Conclusions

Mastitis is widespread in lactating dromedary camels in Banadir and Lower Shabelle, south-central Somalia, with CMT positivity more frequent than overt clinical disease. The predominance of staphylococci, the rise in SCC across CMT categories, and the associations of peri-urban production and teat lesions with CMT positivity indicate that mastitis is biologically important and management-sensitive. Peri-urban herds require intensified milking-hygiene and udder-health interventions, while pastoral herds also require targeted nutritional and herd-health support because poor body condition affected 44.8% of pastoral camels. The detection of antibiotic residues in a selected subset further supports prudent antimicrobial use and milk-safety monitoring. Routine screening, teat-health management, improved nutrition, and context-specific interventions are needed across both production systems. 

## Supplementary Information

Supplementary Material 1.

## Data Availability

The datasets analysed during the current study are available from the corresponding author on reasonable request.

## Abbreviations

AOR: Adjusted odds ratio
AMR: Antimicrobial resistance
BCS: Body condition score
CI: Confidence interval
CMT: California Mastitis Test
CNS: Coagulase-negative staphylococci
DIM: Days in milk
GEE: Generalized estimating equation
IQR: Interquartile range
OR: Odds ratio
SCC: Somatic cell count
SD: Standard deviation
SE: Standard error
TriSensor: Multi-residue screening test
