# Supplementary material for: Prevalence, bacterial etiology, and risk factors of clinical and subclinical mastitis in dromedary camels (Camelus dromedarius) across pastoral and peri-urban production systems in south-central Somalia
Source: BMC Vet Res. 2026 Aug 4;22:650. doi: 10.1186/s12917-026-05792-2 (PMC13729643; doi:10.1186/s12917-026-05792-2)
Supplement: Supplementary file 1 [file 12917_2026_5792_MOESM1_ESM.docx]

**Supplementary Tables**

# *Manuscript:* Prevalence, Bacterial Etiology, and Risk Factors of Clinical and Subclinical Mastitis in Dromedary Camels (*Camelus dromedarius*) Across Pastoral and Peri-Urban Production Systems in Somalia

**Supplementary tables**

**Supplementary Table S1. Study districts by production system (n = 384).**

| **District** | **Overall n(%)** | **Pastoral n(%)** | **Peri-urban n(%)** | **p-value** |
| --- | --- | --- | --- | --- |
| Afgooye | 90 (23.4%) | 90 (40.4%) | 0 (0.0%) | 0.000 |
| Dharkenley | 64 (16.7%) | 0 (0.0%) | 64 (39.8%) |  |
| Garasbaley | 48 (12.5%) | 0 (0.0%) | 48 (29.8%) |  |
| Kaxda | 49 (12.8%) | 0 (0.0%) | 49 (30.4%) |  |
| Walan weyn | 66 (17.2%) | 66 (29.6%) | 0 (0.0%) |  |
| Yaaqbariweyne | 67 (17.4%) | 67 (30.0%) | 0 (0.0%) |  |

**Supplementary Table S2. Camel age group, parity, and lactation stage by production system (n = 384).**

| **Variable** | **Category (clarified)** | **Overall n(%)** | **Pastoral n(%)** | **Peri-urban n(%)** | **p-value** |
| --- | --- | --- | --- | --- | --- |
| Camel age group | <2 years (young) | 46 (12.0%) | 29 (13.0%) | 17 (10.6%) | 0.561 |
|  | 2–5 years (sub- adult/young adult) | 135 (35.2%) | 81 (36.3%) | 54 (33.5%) |  |
|  | >5 years (adult) | 203 (52.9%) | 113 (50.7%) | 90 (55.9%) |  |
| Parity (number of calvings) | 1 calving | 126 (32.8%) | 73 (32.7%) | 53 (32.9%) | 0.360 |
|  | 2–3 calvings | 121 (31.5%) | 76 (34.1%) | 45 (28.0%) |  |
|  | ≥4 calvings | 137 (35.7%) | 74 (33.2%) | 63 (39.1%) |  |
| Lactation stage | Early lactation (as reported) | 89 (23.2%) | 61 (27.4%) | 28 (17.4%) | 0.018 |
|  | Mid lactation (as reported) | 189 (49.2%) | 97 (43.5%) | 92 (57.1%) |  |
|  | Late lactation (as reported) | 106 (27.6%) | 65 (29.1%) | 41 (25.5%) |  |

**Supplementary Table S3. Body condition score and breed/ecotype distribution by production system (n = 384).**

| **Variable** | **Category (clarified)** | **Overall n(%)** | **Pastoral n(%)** | **Peri-urban n(%)** | **p-value** |
| --- | --- | --- | --- | --- | --- |
| Body condition score (BCS) | ≤2.5 (poor) | 124 (32.3%) | 100 (44.8%) | 24 (14.9%) | 0.000 |
|  | 3.0 (moderate) | 129 (33.6%) | 74 (33.2%) | 55 (34.2%) |  |
|  | ≥3.5 (good) | 131 (34.1%) | 49 (22.0%) | 82 (50.9%) |  |
| Breed/ecotype | Hoor | 135 (35.2%) | 91 (40.8%) | 44 (27.3%) | 0.046 |
|  | Aydimo | 91 (23.7%) | 48 (21.5%) | 43 (26.7%) |  |
|  | Gellab | 87 (22.7%) | 44 (19.7%) | 43 (26.7%) |  |
|  | Siifdacar | 71 (18.5%) | 40 (17.9%) | 31 (19.3%) |  |

**Supplementary Table S4. Recent antibiotic use and days in milk by production system (n = 384).**

| **Variable** | **Category** | **Overall** | **Pastoral** | **Peri-urban** | **p-value** |
| --- | --- | --- | --- | --- | --- |
| Recent antibiotic use (last 30 days) | No | 345 (89.8%) | 204 (91.5%) | 141 (87.6%) | 0.281 |
|  | Yes | 39 (10.2%) | 19 (8.5%) | 20 (12.4%) |  |
| Days in milk (continuous) | Mean ± SD | 143.66 ± 96.58 | 141.22 ± 100.53 | 147.05 ± 91.02 | 0.274 |

**Supplementary Table S5. Univariate associations between CMT-positive quarters and camel-level factors (n = 1,536 quarters).**

| **Variable** | **Category** | **CMT negative n(%)** | **CMT positive n(%)** | **p-value** |
| --- | --- | --- | --- | --- |
| Production system | Pastoral | 499 (55.9%) | 393 (44.1%) | 0.001 |
|  | Peri-urban | 303 (47.0%) | 341 (53.0%) |  |
| Camel age group | <2 years | 102 (55.4%) | 82 (44.6%) | 0.534 |
|  | 2-5 years | 274 (50.7%) | 266 (49.3%) |  |
|  | >5 years | 426 (52.5%) | 386 (47.5%) |  |
| Parity | 1 | 258 (51.2%) | 246 (48.8%) | 0.120 |
|  | 2-3 | 271 (56.0%) | 213 (44.0%) |  |
|  | >=4 | 273 (49.8%) | 275 (50.2%) |  |
| Lactation stage | Early | 196 (55.1%) | 160 (44.9%) | 0.385 |
|  | Mid lactation | 383 (50.7%) | 373 (49.3%) |  |
|  | Late lactation | 223 (52.6%) | 201 (47.4%) |  |
| Body condition score | <=2.5 | 267 (53.8%) | 229 (46.2%) | 0.243 |
|  | 3.0 | 277 (53.7%) | 239 (46.3%) |  |
|  | >=3.5 | 258 (49.2%) | 266 (50.8%) |  |
| Breed/ecotype | Hoor | 272 (50.4%) | 268 (49.6%) | 0.651 |
|  | Aydimo | 190 (52.2%) | 174 (47.8%) |  |
|  | Gellab | 184 (52.9%) | 164 (47.1%) |  |
|  | Siifdacar | 156 (54.9%) | 128 (45.1%) |  |
| Antibiotic use last 30 days | No | 714 (51.7%) | 666 (48.3%) | 0.307 |
|  | Yes | 88 (56.4%) | 68 (43.6%) |  |

**Supplementary Table S6. Univariate associations between CMT-positive quarters and udder-health indicators (n = 1,536 quarters).**

| **Variable** | **Category** | **CMT negative n(%)** | **CMT positive n(%)** | **p-value** |
| --- | --- | --- | --- | --- |
| Tick severity | Not recorded | 342 (50.9%) | 330 (49.1%) | 0.255 |
|  | Mild | 271 (55.1%) | 221 (44.9%) |  |
|  | Moderate | 139 (48.9%) | 145 (51.1%) |  |
|  | Severe | 50 (56.8%) | 38 (43.2%) |  |
| Teat lesion severity | Not recorded | 574 (54.2%) | 486 (45.8%) | 0.036 |
|  | Mild | 100 (45.5%) | 120 (54.5%) |  |
|  | Moderate | 107 (52.5%) | 97 (47.5%) |  |
|  | Severe | 21 (40.4%) | 31 (59.6%) |  |

**Supplementary Table S7. Univariate associations between CMT-positive quarters and quarter position (n = 1,536 quarters).**

| **Variable** | **Category** | **CMT negative n(%)** | **CMT positive n(%)** | **p-value** |
| --- | --- | --- | --- | --- |
| Quarter sampled | LF | 201 (52.3%) | 183 (47.7%) | 0.495 |
|  | RF | 212 (55.2%) | 172 (44.8%) |  |
|  | LH | 198 (51.6%) | 186 (48.4%) |  |
|  | RH | 191 (49.7%) | 193 (50.3%) |  |
